# The Role of Pre-Existing Diabetes Mellitus on Hepatocellular Carcinoma Occurrence and Prognosis: A Meta-Analysis of Prospective Cohort Studies

**DOI:** 10.1371/journal.pone.0027326

**Published:** 2011-12-21

**Authors:** Wan-Shui Yang, Puthiery Va, Freddie Bray, Shan Gao, Jing Gao, Hong-Lan Li, Yong-Bing Xiang

**Affiliations:** 1 Department of Epidemiology, School of Public Health, Fudan University, Shanghai, China; 2 State Key Laboratory of Oncogene and Related Genes, Shanghai Cancer Institute, Renji Hospital, Shanghai Jiaotong University School of Medicine, Shanghai, China; 3 Department of Epidemiology, Shanghai Cancer Institute, Renji Hospital, Shanghai Jiaotong University School of Medicine, Shanghai, China; 4 University of New England College of Osteopathic Medicine, Biddeford, Maine, United States of America; 5 Section of Cancer Information, International Agency for Research on Cancer, Lyon, France; Johns Hopkins Bloomberg School of Public Health, United States of America

## Abstract

**Background:**

The impact of pre-existing diabetes mellitus (DM) on hepatocellular carcinoma (HCC) occurrence and prognosis is complex and unclear. The aim of this meta-analysis is to evaluate the association between pre-existing diabetes mellitus and hepatocellular carcinoma occurrence and prognosis.

**Methods:**

We searched PubMed, Embase and the Cochrane Library from their inception to January, 2011 for prospective epidemiological studies assessing the effect of pre-existing diabetes mellitus on hepatocellular carcinoma occurrence, mortality outcomes, cancer recurrence, and treatment-related complications. Study-specific risk estimates were combined by using fixed effect or random effect models.

**Results:**

The database search generated a total of 28 prospective studies that met the inclusion criteria. Among these studies, 14 reported the risk of HCC incidence and 6 studies reported risk of HCC specific mortality. Six studies provided a total of 8 results for all-cause mortality in HCC patients. Four studies documented HCC recurrence risks and 2 studies reported risks for hepatic decomposition occurrence in HCC patients. Meta-analysis indicated that pre-existing diabetes mellitus (DM) was significantly associated with increased risk of HCC incidence [meta-relative risk (RR) = 1.87, 95% confidence interval (CI): 1.15–2.27] and HCC-specific mortality (meta-RR = 1.88, 95%CI: 1.39–2.55) compared with their non-DM counterparts. HCC patients with pre-existing DM had a 38% increased (95% CI: 1.13–1.48) risk of death from all-causes and 91% increased (95%CI: 1.41–2.57) risk of hepatic decomposition occurrence compared to those without DM. In DM patients, the meta-RR for HCC recurrence-free survival was 1.93(95%CI: 1.12–3.33) compared with non-diabetic patients.

**Conclusion:**

The findings from the current meta-analysis suggest that DM may be both associated with elevated risks of both HCC incidence and mortality. Furthermore, HCC patients with pre-existing diabetes have a poorer prognosis relative to their non-diabetic counterparts.

## Introduction

Hepatocellular carcinoma (HCC) is the seventh most common cancer and the fourth leading cause of cancer related death in the world [1]. More than 80% of HCC cases develop in Asian and African countries with 55% of the cases reported in China alone [2], In contrast, the incidence of HCC in the United States and Western Europe is relatively low. These geographical variations are in part explained by variations in the prevalence of chronic infection with hepatitis B virus (HBV) and/or hepatitis C virus (HCV). Although most HCC cases occur in sub-Saharan Africa and Eastern Asia, HCC incidence has been declining in some of these high-rate areas [3]–[5], partly due to universal vaccination against hepatitis B virus in the newborns [3], [5]. On the contrary, the incidence trends of HCC have been increasing over the past three decades in low-endemic areas including the United States, Canada, and Western Europe. In the United States, for instance, the age-adjusted incidence of HCC has recently more than tripled, from 1.6/100,000 in 1975 to 4.9/100,000 in 2005 [6]. The cause of this increase in low-rate areas is not well understood but may reflect the changing patterns of HCC etiology. Although chronic hepatitis C viral infection may explain up to 50% of this increase, HBV infection and alcoholic liver disease (ALD) is unlikely to explain the remainder [7], [8]. In general, more than 25% of HCC cases do not have any known etiology [9], suggesting other risk factors, aside from the recognized factors (HBV, HCV and alcohol), may play an important role in HCC development.

Over the last few decades, the prevalence of diabetes mellitus (DM) has increased substantially and is highly suspected to be associated with an increased risk of HCC. Diabetes mellitus is mainly composed of type I and type II diabetes. Although most available data do not distinguish between the two types, type II diabetes makes up the majority of cases worldwide. In the United States, DM is the sixth leading cause of death and its crude prevalence in adult U.S. population rose from 5.1% in 1988–1994 to 7.7% in 2005–2006 [10]. Many studies, including several case-control studies [11]–[16] and cohort studies [17]–[25], have reported a positive association between DM and HCC risk. A possible explanation for this association relates to the fact that diabetes often occurs as part of the metabolic syndrome itself characterized by a group of biochemical abnormalities and associated clinical conditions which include disturbed glucose and insulin metabolism resulting in hyperglycemia and hyperinsulinemia, dyslipidemia, and hypertension. The metabolic derangements associated with metabolic syndrome (hyperinsulinemia, hyperglycemia, and dyslipidemia) can lead to diabetes mellitus and/or atherosclerotic cardiovascular disease. Moreover, these aforementioned metabolic abnormalities may contribute to the increasing risk of nonalcoholic fatty liver disease (NAFLD), including its most severe form, nonalcoholic steatohepatitis (NASH), and that HCC may be a late subsequent consequence of cirrhosis caused by NAFLD; however some studies have refuted this association [26]–[29].. Additionally, reverse causality is a major concern for causal inference in these case-control studies because in some cases diabetes might itself be a result of cirrhosis.

Diabetes mellitus may be a risk factor for some cancers; however the impact of pre-existing DM on overall cancer prognosis, including cancer recurrence, cancer mortality, and all-cause mortality, remains unclear [30]–[32]. Although diabetes is associated with age-adjusted excess mortality, whether the excess mortality associated with DM in cancer patients is any greater than the excess mortality observed among diabetic patients without cancer requires further investigation. Nonetheless, some studies have reported that pre-existing DM in cancer patients at the time of diagnosis is associated with increased risk of all-cause mortality [30], [31]. In particular, previous studies have shown a significant association between DM and cancer prognosis for specific sites such as breast cancer, prostate cancer, endometrial cancer, colon, and rectum [21], [33]–[38].

There have been several proposed mechanisms explaining the association between DM and cancer prognosis. Type II DM and metabolic syndrome have both been associated with a state of chronic, low grade inflammation. Inflammatory conditions can initiate or promote oncogenic transformation. Concurrently, genetic and epigenetic changes in malignant cells can generate an inflammatory environment which supports tumor progression and hepatocellular carcinoma [39]–[41]. Additionally, DM provides an environment of hyperinsulinemia and hyperglycemia, both of which may increase tumor cell proliferation and metastasis [30], [42]–[43]. Acute exposure to hyperglycemia may increase endothelial cell permeability due to increased generation of reactive oxidative species and structural changes in the basement membrane thereby increasing the likelihood of metastasis [44]–[46]. Also, insulin or insulin like growth factor levels may promote cancer cell and tumor growth [37], [47]–[51]. Furthermore, patients with pre-existing DM often have other diabetes-related comorbid conditions that may influence clinical decisions and response to cancer treatment, including poor response, increased risk of infection and intraoperative morbidity and mortality [36], [52]–[54].

The role of DM on HCC incidence remains controversial and it is less clear whether pre-existing DM can influence overall survival, risk of recurrence, and treatment-related complications in HCC patients. In the limited studies conducted, the impact of DM on hepatocellular carcinoma has been inconsistent with one meta-analysis reporting no significant association with prognosis [31], [32]. We therefore conducted a meta-analysis, combining the results from long-term prospective epidemiological studies, to investigate: i) the association between pre-existing DM and HCC incidence, and ii) the possible effect of pre-existing diabetes mellitus on prognosis in HCC patients.

## Methods

### Searching

We systematically identified studies through searching EMBASE, Medline (PubMed) and the Cochrane Library from their inception to January 1, 2011 for human, English and Chinese-language studies on evaluating the effect of pre-existing diabetes on HCC occurrence and any prognostic outcome in HCC patients. Our overall search strategy included terms for hepatocellular carcinoma (cancer, liver neoplasm, primary liver cancer, hepatocellular carcinoma), diabetes (metabolic syndrome, diabetes mellitus, diabetes, hyperglycemia), and study design (cohort studies, follow-up, prospective studies). Furthermore, the cited references of retrieved articles were hand-searched to locate the additional relevant studies.

### Selection

Articles were included into the meta-analysis if they: i.) were prospective studies; ii.) evaluated the association between diabetes and any HCC prognostic outcome or risk of HCC occurrence; iii.) contained original data and iv.) reported a risk estimate (i.e., hazard ratio or relative risk) regarding pre-existing DM to subsequent incidence or any prognostic outcome and its 95% confidence interval (CI) or its standard error (SE). If the publications were duplicated or shared in more than one study, either the most recent publication or the publication with multivariate-adjusted estimates was included.

### Data abstraction

Two of the authors (PV and W-SY) independently evaluated the eligibility of all retrieved studies from the databases and extracted all the relevant data from each study included using a unified data form. The extracted information included in the data form were as follows: study name (together with first author's name and year of publication), country, study design (clinic-based or population-based cohort studies), inclusion for study cohort, sample size (numbers of pre-existing diabetes and cohort size), range of follow-up time, statistical adjustments for confounders in analysis and study results (adjusted RR or HR with their corresponding 95%CIs for HCC occurrence or any HCC prognostic outcome by diabetes), method of diabetes and outcome ascertainment. Afterwards, two lists from evaluators were compared and disagreements were resolved by consensus between the two review authors.

A 7-point scoring system was created to evaluate study quality. Studies that confirmed pre-existing DM by medical record or medication use were assigned 1 point. Studies that used medical records to evaluate the outcome received 2 points, whereas those that used a death certificate or cancer registry received 1 point. Allowing for major potential confounders (i.e., HBV, HCV and alcohol drinking) being controlled in varying degrees across the included articles, studies adjusting for HBV in an African country or an Asian country (except for Japan) or HCV in a European country, USA or Japan received 2 points; those that also adjusted for one or more of the two remaining major potential confounders received an additional 1 point. Clinic-based cohort studies with loss to follow-up of <5% received 1 point, while population-based cohort studies with loss to follow-up of <20% received 1 point. Studies could receive up to maximum score of 7 points. High quality studies were defined as a study with a quality score≥5 points.

### Statistical methods

To fully consider the effect of pre-existing DM in HCC outcome of interest (occurrence and prognosis), the aim of our analysis was divided into 5 parts based on different outcomes reported in each included article. In 28 articles [17]–[29], [55]–[69], 7 [17]–[19], [21]–[23], [61] of 12 [17]–[19], [21]–[23], [56], [58], [61], [63], [64], [68] population-based cohort studies and 7 [20], [24]–[29] of 16 [20], [24]–[29], [55], [57], [59], [60], [62], [65]–[67], [69] clinic-based cohort studies reported the risk of pre-existing DM in HCC occurrence and were included in the first part of meta-analysis on DM in relations to HCC incidence risk. In the second part, 6 [56], [58], [61], [63], [64], [68] population-based cohort studies that provided data for risk of HCC specific mortality in subjects with history of diabetes were analyzed. In the third part, 6 [55], [60], [62], [66], [67], [69] clinic-based cohort studies were summarized to evaluate the prognostic effect of pre-existing DM on all-cause mortality in HCC patients. We statistically combined 4 [57], [59], [67], [69] and 2 [62], [65] clinic-based cohort studies for the fourth and fifth part of our investigation to evaluate the association between the pre-existing DM with HCC patients' prognosis on recurrence-free survival (RFS) and cancer treatment-related hepatic decomposition (HD) occurrence, respectively. To compute a summary RR with its 95% CI, we used the study-specific adjusted RR or HR and its 95%CI in all analyses. All RR or HR extractions were performed separately by PV and W-SY. Disagreements were resolved by discussion.

In this analysis, we examined possible heterogeneity in results across studies using the Cochran *Q* and *I^2^* statistics [70]. The null hypothesis that the studies are homogeneous would be rejected if *P*-value for heterogeneity is less than 0.10 or *I^2^*≥50%. When there is significant heterogeneity among study results, the random effects model (DerSimonian and Laird method) [71] was used to calculate summary estimate assuming that the studies included in the meta-analysis have the varying effect size across studies. Otherwise, the summarized estimate was calculated based on the fixed effects model (the inverse variance method), assuming that the studies included in the meta-analysis have the same effect size.

There is a tendency on average to produce results that appear significant, given negative or near neutral results are almost never published. This is the so-called publication bias and may bias results of the meta-analysis. In an attempt to evaluate the possible publication bias, Egger's test (linear regression method) [72] and Begg's test (rank correlation method) [73] were used, and *P*-value<0.05 was considered representative of significant statistical publication bias. If publication bias was identified, the “trim and fill” method, suggested by Duval and Tweetdie [74], was adopted to further assess the effect of correcting the publication bias. This method relies on scrutiny of one side of a funnel plot for asymmetry assumed due to publication bias and recalculates a pooled estimate considering the number of studies missing from a meta-analysis so that the funnel plot is more symmetric. All data analyses were performed using the R 2.12.1 statistical software (meta 1.6-1 package) (R Development Core Team, 2010, available from: www.r-project.org).

## Results

### Literature search and quality assessment

**Figure 1:**
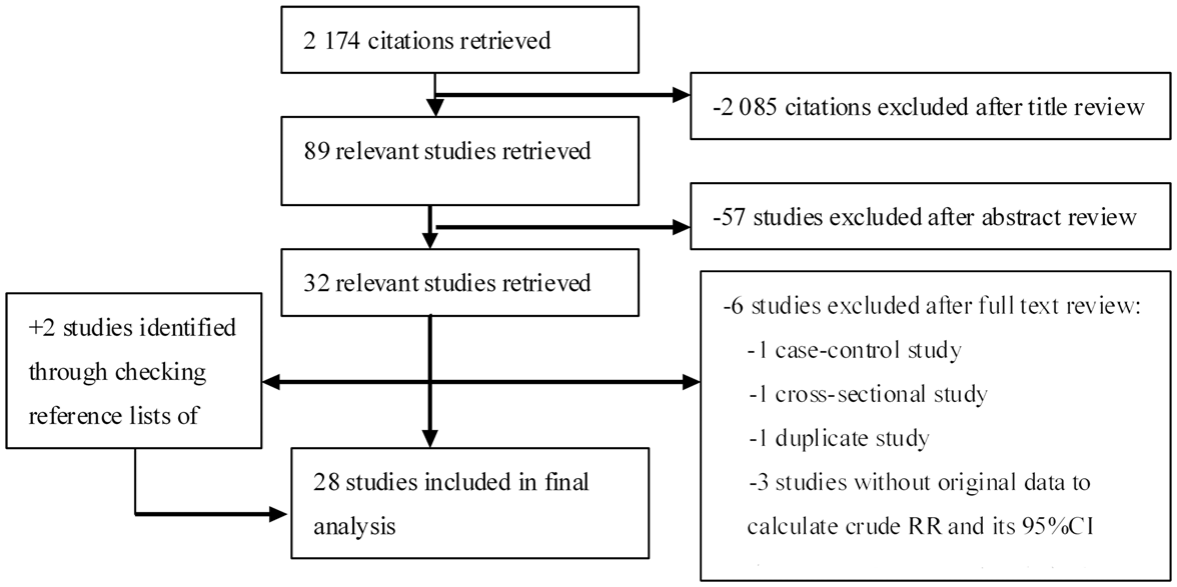
Selection of studies in the Meta-analysis.

Figure 1 shows a flow diagram of how we selected relevant studies. Our systematic literature search yielded a total of 28 articles in the final analysis, comprising 12 population-based cohort studies and 16 clinic-based cohort studies. Outcomes reported in each article included HCC occurrence (n = 14) [17]–[29], [61], HCC-specific mortality (n = 6) [56], [58], [61], [63], [64], [68], all-cause mortality (n = 6) [55], [60], [62], [66], [67], [69], recurrence-free survival (n = 4) [57], [59], [67], [69], and hepatic decomposition as a complication (n = 2) [62], [65]. All included studies were published between 1998 and 2010, of which 64% (n = 18) were published in 2005 or more recent years. The studies were conducted in the following regions: Japan (n = 9) [20], [22], [23], [25], [57]–[59], [68], [69], China (n = 8) [18], [21], [26], [55], [62], [65]–[67],Korea (n = 2) [60], [61], USA (n = 3) [24], [29], [63], Europe (n = 4) [19], [27], [28], [64], Israel (n = 1) [17] and other regions (n = 1) [56]. The cohort ranged in size from 40 [57] to 1,298,385 [61].The duration of follow-up ranged from 2.78 years [21] to 25 years [64] in population-based cohort studies and ranged from 18 months [55] to 7 years [8] in clinic-based cohort studies. Seven studies [18], [19], [21], [26], [55], [66], [68] assessed type II DM only; and an additional 21 studies did not distinguish between type I and type II DM. The characteristics of the included studies are shown in Table S1.

According to our 7-point scoring system, the study-specific quality scores are summarized in Table 1. Half (n = 14) of the studies were defined as high quality studies (score≥5 points), of which 12 were clinic-based cohort studies and 2 were population-based prospective studies. Of the 28 studies, 19 [17], [18], [20], [21], [24]–[27], [29], [55]–[57], [59], [61], [62], [65]–[67], [69] used medical records or documented use of diabetic medicine to ascertain DM diagnosis and the remaining 9 studies used self-reported data or a disease registry. Medical records were used to ascertain outcomes in 18 [20]–[25], [27]–[29], [55]–[57], [59], [62], [65]–[67], [69] studies while others used cancer registry data or death certificate.

**Table 1:** Assessment of study quality.

Study	Diabetes	Outcome	Loss to follow-up		Major confounders control			Quality
	ascertainment	ascertainment	<20%(PC)	<5%(HC)	HBV	HCV	Alcohol drinking	score
Ikeda Y 1998 [69]	MR or MU	MR	-	Yes	Yes	Yes	No	
Fujino Y 2001 [68]	SR	DC	Unknown	-	No	No	Yes	1
Tazawa J 2002 [25]	MR or MU	MR	-	Yes	No	Yes	No	6
Poon RTP 2002 [67]	MR or MU	MR	-	Yes	Yes	No	Yes	7
Huo TI 2003 [66]	MR or MU	MR	-	Yes	Yes	Yes	No	7
Huo TI 2003 [65]	MR or MU	MR	-	No	Yes	No	No	5
El-Serag HB 2004 [24]	MR	MR	-	Yes	No	No	No	3
Huo TI 2004 [62]	MR or MU	MR	-	Yes	Yes	No	No	6
Coughlin SS 2004 [63]	MR or SR	CR	Unknown	-	No	No	Yes	1
Batty GD 2004 [64]	SR	CR	Unknown	-	No	No	No	1
Jee SH 2005 [61]	MR	CR	Unknown	-	No	No	Yes	2
Park SM 2006 [60]	MR or SR	CR	-	Yes	No	No	Yes	2
Inoue M 2006 [23]	SR	MR	Yes	-	No	No	Yes	3
Khan M 2006 [22]	SR	CR	Unknown	-	No	No	Yes	1
Lai MS 2006 [21]	MR	MR	Unknown	-	Yes	Yes	Yes	6
Torisu Y 2007 [20]	MR	MR	-	No	No	No	Yes	3
Komura T 2007 [59]	MR	MR	-	Yes	Yes	Yes	Yes	7
Ioannou GN 2007 [29]	MR	MR	-	Yes	Yes	Yes	Yes	7
Kawamura Y 2008 [57]	MR	MR	-	No	No	No	Yes	3
Di Costanzo GG 2008 [28]	MU or SR	MR	-	Yes	No	No	Yes	5
Veldt BJ 2008 [27]	MR or MU	MR	-	Yes	No	No	Yes	6
Oba S 2009 [58]	SR	CR	Unknown	-	No	No	Yes	1
Ogunleye AA 2009 [19]	DR	CR	Unknown	-	No	No	No	1
Wang CS 2009 [18]	MR	CR	Yes	-	Yes	Yes	No	7
Lam EK 2010 [56]	MR	Unknown	Unknown	-	No	No	No	1
Chodick G 2010 [17]	MR or MU	CR	No	-	No	No	No	2
Hung CH 2010 [26]	MR	DC	-	Yes	Yes	No	Yes	6
Huo TI 2010 [55]	MR or MU	MR	-	Yes	Yes	Yes	No	7

Abbreviations: CR, cancer registry; DC, death certificate; DR, diabetes registry; HC, hospital-based cohort study; MR, medical record; MU, medication use; PC, population-based cohort study; SR, self-report.

### Diabetes and HCC incidence

**Figure 2:**
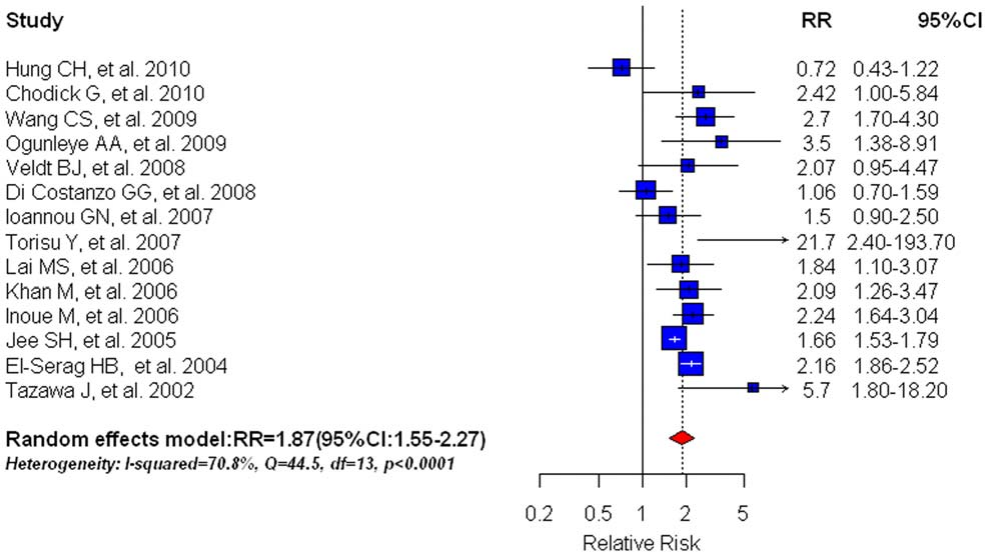
Summary estimate of relative risk (RR) for HCC incidence in diabetic patients in comparison with non-diabetic patients. Squares represent study-specific estimates (size of the square reflects the study-specific statistical weight, i.e. inverse of the variance); horizontal lines represent 95% CIs; diamonds represent summary estimates with corresponding 95% CIs.

Based on 14 studies, pre-existing DM was associated with an 87% risk increase for HCC incidence (RR = 1.87, 95%CI: 1.55–2.27; shown in Figure 2). Statistically significant heterogeneity was found among these studies (I^2^ = 70.8%, Q = 44.5, df = 13, P<0.0001; Table 2) and thus random effects models were employed. There was no indication of a publication bias, either from Egger's test (P = 0.250) or from Begg's test (P = 0.295).

**Table 2:** Meta-analysis for pre-existing diabetes mellitus and HCC incidence.

	No. of	Meta-RR	Heterogeneity	
	studies	(95%CI)	*I^2^* (%)	*P*-value
**Overall**	14	1.87(1.55–2.27)	70.8	<0.0001
**Higher quality (score≥5)**	7	1.66(1.09–2.51)	73.8	0.0008
**Study design**				
Clinic-based cohort	7	1.75(1.10–2.79)	81.7	<0.0001
Population-based cohort	7	2.04(1.67–2.48)	43.6	0.1005
**Major confounders control**				
Yes	7	2.05(1.23–3.42)	75.1	0.0005
No	7	1.88(1.54–2.30)	70.5	0.0024
**Study areas**				
Japan	4	2.80(1.66–4.68)	53.7	0.0903
Asian countries (except for Japan)	4	1.59(1.06–2.38)	78.9	0.0026
European Union+USA	5	1.77(1.23–2.57)	68.6	0.0126
Israel	1	2.42(1.00–5.84)	-	-
**Year of publication**				
≤2005	3	2.00(1.50–2.66)	84.7	0.0014
>2005	11	1.85(1.37–2.51)	68.1	0.0005
**Diabetes ascertainment**				
MR or MU	10	1.88(1.49–2.37)	73.3	<0.0001
Others	4	1.90(1.12–2.97)	71.6	0.0143
**Outcome ascertainment**				
MR	8	1.95(1.48–2.58)	64.4	0.0063
Others	6	1.83(1.28–2.61)	72.2	0.0030

MR, medical record; MU, medication use; Meta-RR, Meta-relative risk.

In the subgroup analyses (Table 2), when analyses was restricted to high quality studies only, we observed 21% reduction in risk estimate as compared with the overall estimate (RR = 1.66, 95%CI: 1.09–2.51; n = 7). By study design, we found similar results for clinic-based cohorts (RR = 1.75, 95%CI: 1.10–2.79) and for population-based cohorts (RR = 1.73, 95%CI: 1.61–1.86). Diabetes was associated with increased risk of HCC occurrence in studies with (RR = 2.05, 95%CI: 1.23–3.42) and without (RR = 1.88, 95%CI: 1.54–2.30) adjustments for major confounding factors.. Analyses stratified by study area showed that DM was associated with a greater risk of HCC in Japan (RR = 2.80, 95%CI: 1.66–4.68; n = 4) than in other Asian countries (RR = 1.59, 95%CI: 1.06–2.38; n = 4). Analyses confined to studies using medical records or medication use as a means of DM ascertainment yielded similar results (RR = 1.88, 95%CI: 1.49–2.37; n = 10) compared to studies using self-report data or disease registry to determine DM status (RR = 1.90, 95%CI: 1.12–2.97; n = 4). Studies using medical records to ascertain outcome (RR = 1.95, 95%CI: 1.48–2.58; n = 10) demonstrated slightly higher increased risk than studies adopting other methods for outcome ascertainment (RR = 1.83, 95%CI: 1.289–2.61; n = 6). When examining differences over time, we found that studies published after 2005 had a summary estimate with a meta-RR of 1.85(95%CI: 1.37–2.51), while studies published before 2005 had a summary estimate with a meta-RR of 2.00(95%CI: 1.50–2.66).

### Diabetes and HCC specific mortality

**Figure 3:**
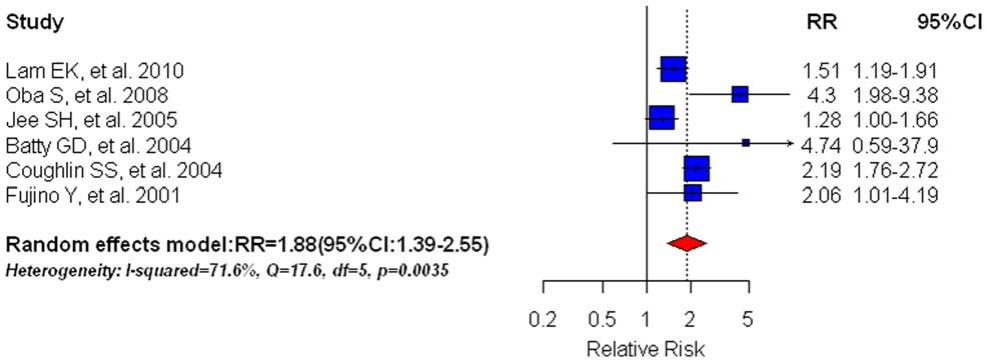
Summary estimate of relative risk (RR) for HCC specific mortality in diabetic patients compared with their non-diabetic counterparts. Squares represent study-specific estimates (size of the square reflects the study-specific statistical weight, i.e. inverse of the variance); horizontal lines represent 95% CIs; diamonds represent summary estimates with corresponding 95% CIs.

Six population-based prospective studies [56], [58], [61], [63], [64], [68] on HCC specific mortality were included and indicated that pre-existing DM was associated with a 1.88-fold elevated risk (95%CI: 1.39–2.55; shown in Figure 3) of HCC-specific mortality. We found no statistical evidence of publication bias (Egger's test: P = 0.371; Begg's test: P = 0.851). The random effect model was implemented due to substantial heterogeneity in the estimates across studies (I^2^ = 71.6%, Q = 17.6, df = 5, P = 0.0035). The meta-RR was 2.18(95%CI: 1.77–2.68) in two studies [63], [68] that controlled for major confounders and 1.76(95%CI: 1.18–2.63) in 4 studies [56], [58], [61], [64] that did not control for major confounders; Studies published prior to 2005 had a meta-RR of 2.20(95%CI: 1.78–2.70; n = 3) while studies published after 2005 had a meta-RR of 1.70(95%CI: 1.13–2.55). Additionally, studies using medical records or medication use as DM ascertainment had a meta-RR of 1.40(95%CI: 1.18–1.66; n = 2) whereas studies using other methods to confirm DM yielded a meta-RR of 2.35(95%CI: 1.83–3.02; n = 4).

### Diabetes and all-cause mortality

**Figure 4:**
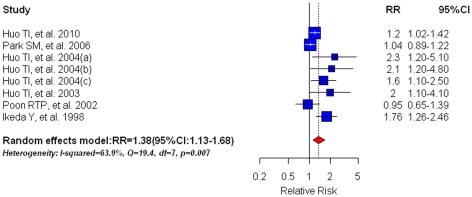
Summary estimate of relative risk (RR) for all-cause mortality in HCC patients with pre-existing diabetes mellitus compared with their non-diabetic counterparts. Squares represent study-specific estimates (size of the square reflects the study-specific statistical weight, i.e. inverse of the variance); horizontal lines represent 95% CIs; diamonds represent summary estimates with corresponding 95% CIs.

Six studies [55], [60], [62], [66], [67], [69] assessing the association between DM and all-cause mortality were collapsed to obtain a meta-RR of 1.38(95%CI: 1.13–1.68)(Figure 4), suggesting a poor overall survival in HCC patients with pre-existing diabetes compared with their non-diabetic counterparts. However, large heterogeneity existed in these studies (I^2^ = 63.9%, Q = 19.4, df = 7, P = 0.0007); hence the random effect model was adopted. We observed significant publication bias according to both Egger's test (P = 0.048) and Begg's test (P = 0.030). The trim and fill method was used to recalculate the meta-RR thus correcting for the publication bias. The adjusted risk estimate however could not reverse this significant positive association (RR = 1.22, 95%CI: 1.00–1.49; P = 0.0492).

Subgroup analyses confined to high quality studies showed a strong RR for all-cause mortality in HCC patients (RR = 1.49, 95%CI: 1.18–1.87; n = 5). When analysis was restricted to HCC patients who received curative surgery only [62], [66], [67], [69], the meta-RR was 1.64(95%CI: 1.15–2.33); Studies controlling for major confounders yielded a RR of 1.49(95%CI: 1.18–1.87; n = 5). Studies using medical records or medication use as diabetes ascertainment had a meta-RR of 1.49(95%CI: 1.18–1.87; n = 5). In our sensitivity analysis, excluding the estimate by Huo et al 2004(a) [62] resulted in the lowest summary estimate (RR = 1.32, 95%CI:1.09–1.61) whereas omission of the study by Park et al [60] resulted in the highest summary estimate (RR = 1.49, 95%CI:1.18–1.87).

### Diabetes and recurrence-free survival in HCC patients

**Figure 5:**
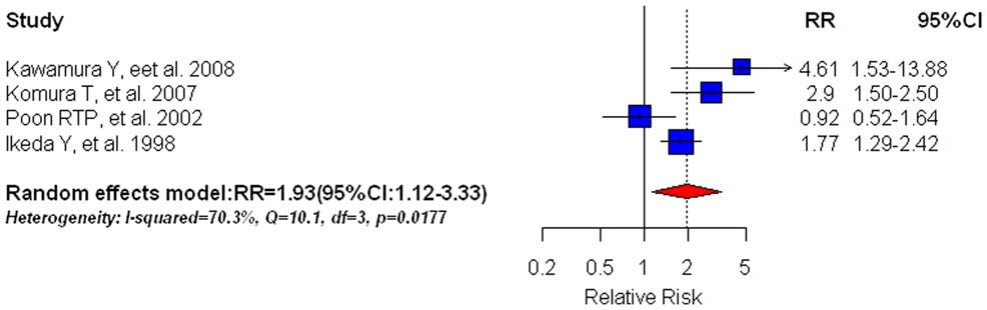
Summary estimate of relative risk (RR) for HCC recurrence-free survival (RFS) in HCC patients with pre-existing diabetes mellitus in comparison with non-diabetic patients. Squares represent study-specific estimates (size of the square reflects the study-specific statistical weight, i.e. inverse of the variance); horizontal lines represent 95% CIs; diamonds represent summary estimates with corresponding 95% CIs.

Of the 4 [57], [59], [67], [69] articles that reported pre-existing DM and recurrence-free survival in HCC patients, our meta-analysis identified pre-existing DM as a significant predictor for HCC recurrence (RR = 1.93, 95%CI: 1.12–3.33)(Figure 5). We found no evidence of publication bias with either Egger's test (P = 0.174) or Begg's test (P = 0.625). Due to substantial heterogeneity across studies (I^2^ = 70.3%, Q = 10.1, df = 3, P = 0.0177), we used the random effect model. Sensitivity analysis showed that removing the study by Poon RTP et al [67] had a minimal effect on the summary estimate (RR = 2.05, 95%CI: 1.56–2.70). When analysis was restricted to HCC patients receiving curative surgery only, we found that the recurrence risk in diabetic patients was 1.66(95%CI: 0.96–2.87) compared to non-diabetic patients.

### Diabetes and hepatic decomposition as a complication in HCC patients

**Figure 6:**
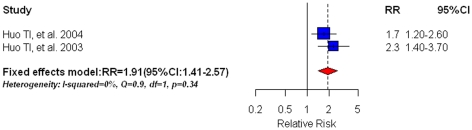
Summary estimate of relative risk (RR) for hepatic decomposition (HD) occurrence in HCC patients with pre-existing diabetes mellitus in comparison with non-diabetic patients. Squares represent study-specific estimates (size of the square reflects the study-specific statistical weight, i.e. inverse of the variance); horizontal lines represent 95% CIs; diamonds represent summary estimates with corresponding 95% CIs.

We combined 2 studies [62], [65] on DM and hepatic decomposition (HD) as a complication in HCC patients. Subjects with diabetes had a significantly increased risk of HD occurrence, compared with non-diabetic subjects (RR = 1.91, 95%CI: 1.41–2.57) (shown in Figure 6). We did not detect any significant heterogeneity. Furthermore, testing publication bias was impossible, given the limited number of studies included.

## Discussion

In our meta-analysis of HCC incidence, DM was significantly associated with an 87% elevated risk of HCC occurrence. This positive association was not reversed across subgroup analyses, regardless of study quality, study area, study design, statistical adjustments, and year of publication (shown in Table 2). Interestingly, we found a higher liver risk of cancer incidence associated with DM in Japan (HCV-related HCC area) than in other Asian countries (HBV-related HCC areas). This geographic variability may reflect differences in etiology and need to be further explored. Due to limited information available regarding effect modification between DM and other important risk factors in relation to HCC incident risk, we were not able to evaluate the possible interactions. Moreover, the results of the subgroup analyses by diabetes and outcome ascertainment were consistent with the comprehensive meta-analysis, supporting the argument that self-reported history of diabetes may be reasonably accurate [68], [75], [76].

Compared with incidence studies, mortality studies have less superiority in causal inference, especially in DM and HCC studies. Given the long latent time period between DM onset and HCC death, it is impossible that the relatively limited follow-up period is sufficient to clarify the effects of reverse causality. Furthermore, this relatively short duration of follow-up might not capture all mortality from HCC cases with longer survival time. Additionally, most population-based studies on HCC mortality ascertained death from HCC based on national vital statistics, where DM related death or associated death may not always be recorded on death certificates among cancer cases [77]; hence this approach seemed to be unreliable. The combined result for HCC specific mortality of the 6 population-based studies, however, was similar to that for HCC incidence (HR = 1.88, 95%CI: 1.39–2.55). This close similarity could be explained by long duration of follow-up, high quality of cancer death registries in the included mortality studies, and high HCC case-fatality of incident cases. Among those studies, the follow-up time ranged from 4 years to 25 years where 4 of the 6 studies reported more than 10 years of follow-up [61], [63], [64], [68]. Subgroup analyses showed that combined estimates for DM-associated HCC mortality varied across selected strata of different methodology of diabetes measurement, major confounder adjustment, and year of publication. These discrepancies may be partly due to the small number of studies within each stratum.

Despite the consistent findings from HCC incidence and specific mortality studies, several issues relating to casual inference on the association between DM and HCC should be noted. First, although it is almost certain that the diagnosis of diabetes preceded the diagnosis of HCC in cohort studies we analyzed, the possible reverse causality in some studies could not be ruled out because it was unknown as to whether diabetes preceded the underlying chronic liver disease, and in some cases diabetes might be caused by these chronic liver disease. In studies of DM and HCC incidence and specific mortality, only one [65] excluded patients with known baseline liver disease from the cohort entry. Second, cohort studies in the analyses that had a relatively short duration of follow-up and/or examined younger populations were not well suited to evaluate the temporality, given the low incident HCC cases or power, and greater loss to follow-up in these studies. For example, two hospital-based [27], [29] and three population-based [19], [21], [56] cohort studies reported an average follow-up period of no more than 4 years, however the time of follow-up in other studies was fairly long (>4years). Third, although most included studies considered major confounding factors such as HBV and HCV infection, body mass index, and alcohol drinking, the inability to adjust fully for other important risk factors, particularly for treatment modalities for diabetes, could have biased the results. Evidence show that some medications for diabetes such as metformin [78] can decrease the risk of cancer, whereas use of exogenous insulin and insulin secretagogues such as sulfonylureas can increase the risk of cancer incidence and/or mortality [79], [80]. Lastly, the duration of diabetes at cohort entry is less clear across the studies where only one study [61] evaluated a duration-response relationship (changes in RR for HCC according to different durations of diabetes). Consequently, we failed to evaluate such duration-response trends in our analysis and thus cannot draw a firm conclusion. Therefore, additional studies are warranted to better define the onset of diabetes in relationship to onset of liver disease, and to clarify how any excess risk conveyed by diabetes is mediated by duration and treatment modalities of diabetes.

Although reported associations between pre-existing DM and mortality in patients with cancer have been inconsistent and varied with site, our results indicate that DM is associated with cancer prognosis. Having pre-existing DM increased the risk of all-cause mortality, recurrence after HCC treatment, and hepatic decomposition. The risk of all-cause mortality increased by 38% in patients with diabetes compared to those without diabetes. The magnitude of association only increased when analysis was restricted to high quality studies (49%) and remained consistent amongst studies adjusting for major confounders. Although our results indicate that pre-existing DM portends an elevated all-cause mortality, it is important to note that these data do not necessarily suggest a causal relationship. Such elevated risk could be associated with DM due to increased risk of complications, morbidity, and mortality associated with diabetes itself. We were unable to analyze the data further to assess mortality risks excluding DM-related causes of mortality because most of the studies involved in the analysis assessed overall survival and all-cause mortality. Of the six studies analyzed, only one study confined analysis to cancer related deaths, excluding DM related causes of death [60], in which the authors found that a positive association remained between DM and cancer mortality with or without the inclusion of DM-related deaths. Additionally, poor prognosis amongst patients with pre-existing DM may be attributable to a multitude of interactions and factors. These factors include tumoral factors such as size, extent of liver damage/cirrhotic factors, tumor recurrence, and DM-associated factors such as insulin intolerance [62]. One study found that DM was a poor prognostic indicator of long-term survival in patients with tumors <5 cm due to the occurrence of DM-related deaths [62]. Moreover, most patients with HCC have liver cirrhosis as a result of long term chronic liver disease. Diabetes may accelerate mortality by accelerating liver fibrosis, inflammation with increased inflammatory markers and cytokines resulting in severe liver failure [81]–[83] and poor cancer prognosis [39]–[41]. Also, it is possible that pre-existing diabetes may potentiate the incidence of bacterial infections in cirrhotic patients which has been shown to increase mortality [84]. However, the pathophysiology underlying cancer prognosis and diabetes remains uncertain and requires further investigation [32]. It is important to note that our analysis of hepatic decomposition was limited to few studies and should be interpreted with caution.

We observed that the risk estimate for HCC specific mortality was higher than all-cause mortality in patients with pre-existing diabetes. The elevated mortality risk may be attributed to risk related to HCC treatment and not necessarily due to the natural progression of HCC. The etiology of HCC is complex and influences treatment options available. Although treatment vary world-wide, first line treatment of early stage HCC is surgical resection, liver transplantation, and ablative therapies all with curative intent. For patients with intermediate stage disease with multifocal lesions and without vascular invasion, the treatment option is transcatheter arterial chemoembolization (TACE) [85]–[87]. Theoretically, liver transplantation is ideal because it removes the tumor along with accompanying liver disease. Due to a shortage of donor livers and long waiting times for transplantation, most clinicians advocate for surgical resection. However, complications of surgery include decreased liver function, inadequate liver remnant and hepatic decomposition, all of which may impact prognosis. Additionally, patients with pre-existing DM and HCC have liver cirrhosis and thus decreased liver function, and/or other diabetes related comorbidities making them poor candidates for surgery or more aggressive treatments resulting in a worse prognosis [53], [86]. Overall, it is difficult to distinguish death from treatment related liver failure, other treatment related complications, diabetes or HCC. Analyzing studies across time periods, we found that mortality risks have declined when comparing current studies with earlier studies. This slight decline may be a result of improvements with diabetes management, glucose control [88], [89] as well as HCC classification, identification of therapeutic targets, and prognosis [85], [87], [90].

Although diabetes was found to be significantly associated with recurrence of HCC after treatment, when restricted to patients who received curative surgery only, the significant association disappeared. The attenuation of risk may result from inherent characteristics of surgery. It is commonly accepted that HCC recurrence is not a result of inadequate resection but more a result of microscopic tumor foci or due to microscopic dissemination of neoplastic cells during surgical procedures [87], [91], [92].

It remains unclear whether diabetes is directly associated with mortality in cancer patients, if it's more of an underlying biologic factor that alters cancer risk such hyperinsulinemia, or whether the cancer-diabetes association is indirect and a result of common risk factors such as obesity. In order to better understand the relationship, it is important to consider levels of insulin, glucose, and other diabetes related biomarkers such as adiponectin. Also, it is pertinent to understand duration of disease and disease management as these factors may also impact diabetes and/or cancer prognosis and outcome [78]–[80]. In addition to pre-existing DM and associated DM comorbidities, other influences on HCC prognosis and HCC treatment response may also include the treatment and management of DM itself. Diabetic treatments may influence HCC prognosis by creating an environment of hyperinsulinemia. One study found insulin therapy for diabetic patients with advanced HCC resulted in a higher recurrence after hepatic resection [59]. Similarly, sulfonylurea agents provide glycemic control but also create an environment of hyperinsulinemia [93]. Thus, high insulin, rather than high glucose, may be an important contributing factor of HCC progression and impact how cells respond to HCC treatment [94].

The key strength of our meta-analysis is that our results were based on cohort studies, thus ensuring that DM diagnosis preceded the hepatocellular carcinoma and have less recall bias due to its prospective nature. Nonetheless, some limitations should be mentioned. First, substantial heterogeneity was found across the component studies. This was partly because of different study areas, study designs, statistical adjustments and methods of diabetes and outcome assessment in each study according to subgroup analyses. However, heterogeneity still existed in many subgroups, indicating that other factors may explain this heterogeneity. Second, publication bias was detected in the meta-analysis of all-cause mortality, however, the adjusted estimate based on trim and fill method had a slightly decrease and could not reverse the significant positive association, and no publication bias was found in other parts.

### Implications and conclusions

In this meta-analysis, we found an increased risk of HCC in patients with diabetes mellitus. This finding underscores the need for preventative measures of diabetes management including weight control, promotion of measures to increase physical activity, and maintenance of a healthy diet. We also found that pre-existing DM is associated with adverse outcomes in hepatocellular carcinoma throughout its entire proceeding, from occurrence, progression, and to mortality. While the mechanism underlying the association between DM and prognosis remains unclear, it is important to monitor patients for post-operative recurrence, post-operative complications and hepatic decomposition.

Future studies should therefore 1) investigate how preexisting diabetes influences clinical decisions and how patients with DM diagnosed with HCC respond to varying treatment modalities for the latter; 2) determine the role of DM treatment in response to HCC treatment and prognosis, and 3) clarify the pathophysiology underlying liver cancer prognosis and diabetes. In addition, the effect of treatments and duration of diabetes should be taken into account in future etiological research.

## Supporting Information

Table S1 **Characteristics of studies included in the meta-analysis (DOC).** ALT,alanine transferase; AST, aspartic transaminase; AFP,α-fetoprotein; BMI, body mass index; BP, blood pressure, CI, confidence interval; CT, computed tomography imaging; CTP, Child-Turcotte-Pugh; d, day; DCP, des-gamma carboxyprothrombin; DM, diabetes mellitus; ECOG, Eastern Cooperative Oncology Group performance status; FEV1, forced expiratory volume in one 1 second; Hb, hemoglobin; HBV, hepatitis B virus; HbA1c, hemoglobin A1c; HbsAg, hepatitis B surface antigen; HC, hospital-based cohort; HCC, hepatocellular carcinoma; HCV, hepatitis C virus; HIV-1, human immunodeficiency virus 1; HR, hazard ratio; ICG-R15, indocyanine green retention rate at 15 minutes; IFN, interferon; INR, international normalized ratio; m, month; MRI, magnetic resonance imaging; MV, multivariate analysis; SES, socioeconomic status; PA, physical activity; PC, population-based cohort; PT, prothrombin time; PV, portal vein; RFA, radio frequency ablation; TACE, transarterial chemoembolization; UV, univariate analysis; y, year.
(DOC)
