# Supplementary material for: The Role of Pre-Existing Diabetes Mellitus on Hepatocellular Carcinoma Occurrence and Prognosis: A Meta-Analysis of Prospective Cohort Studies
Source: PLoS One. 2011 Dec 21;6(12):e27326. doi: 10.1371/journal.pone.0027326 (PMC3244388; doi:10.1371/journal.pone.0027326)
Supplement: Table S1 — Characteristics of studies included in the meta-analysis (DOC). ALT,alanine transferase; AST, aspartic transaminase; AFP,α-fetoprotein; BMI, body mass index; BP, blood pressure, CI, confidence interval; CT, computed tomography imaging; CTP, Child-Turcotte-Pugh; d, day; DCP, des-gamma carboxyprothrombin; DM, diabetes mellitus; ECOG, Eastern Cooperative Oncology Group performance status; FEV1, forced expiratory volume in one 1 second; Hb, hemoglobin; HBV, hepatitis B virus; HbA1c, hemoglobin A1c; HbsAg, hepatitis B surface antigen; HC, hospital-based cohort; HCC, hepatocellular carcinoma; HCV, hepatitis C virus; HIV-1, human immunodeficiency virus 1; HR, hazard ratio; ICG-R15, indocyanine green retention rate at 15 minutes; IFN, interferon; INR, international normalized ratio; m, month; MRI, magnetic resonance imaging; MV, multivariate analysis; SES, socioeconomic status; PA, physical activity; PC, population-based cohort; PT, prothrombin time; PV, portal vein; RFA, radio frequency ablation; TACE, transarterial chemoembolization; UV, univariate analysis; y, year. (DOC) [file pone.0027326.s001.doc]

Table S1 Characteristics of studies included in the meta-analysis

| Study | Country | Study  design | Inclusion for cohort | No. of  DM/cohort size(%) | Follow-up  Time | Adjustments assessed | Outcome: RR/HR (diabetic patients vs. non-diabetic counterparts ^‡^) |
| --- | --- | --- | --- | --- | --- | --- | --- |
| Ikeda Y, et al. 1998 ^69^ | Japan | HC | 33-79 years  HCC diagnosis undergoing curative hepatic resection. | 87/342  (25) | 1278 d (median) | Age, sex, HBV, HCV, bilirubin, albumin, glutamic oxaloacetic transaminase, glutamic pyruvic transaminase, platelet, ICG-R15, AFP level, max tumor diameter, differentiation, surgical margin, capsular formation, invasion to PV, intrahepatic metastasis, stage | All-cause mortality in HCC patients:   - RR=1.76(95%CI:1.26-2.46);   Recurrence-free survival in HCC patients:   - RR=1.77 (95%CI: 1.29-2.42).   MV: DM, albumin, ICG-15, AFP, poorly differentiated histology, intrahepatic metastasis, and tumor stage. |
| Fujino Y, et al.2001 ^68^ | Japan | PC | 30-79 years  Inhabitants from Fukuoka, details on smoking status and alcohol consumption.  No previous cancer, diabetes < 30 years old, providing a history of diabetes, | 364/7308  (5) | 10.6y(mean) | Age, sex, alcohol, smoking, history of hepatitis, and cirrhosis | HCC specific mortality in both men and women:   - HR=2.06(95%CI: 1.01-4.19). |
| Tazawa J, et al. 2002 ^25^ | Japan | HC | HCV infected patients who undergone liver biopsy under clinical diagnosis of **chronic hepatitis** **without cirrhosis** 1987-1995. Histological findings compatible with chronic viral hepatitis.  No evidence of HCC at enrollment or serious complication | 23/279  (8) | HCC 73.9 m (mean)  No HCC 65.5 m (mean) | Age, sex, habitiual alcohol intake, HCV genotype, viral load, history of blood transfusion, AFP level at entry, histological findings (staging & grading), IFN therapy | HCC incidence:   - RR=5.7(95%CI: 1.8-18.2). |
| Poon RTP, et al. 2002 ^67^ | China | HC | Patients undergoing hepatic resection for HCC^α^ . | 62/525  (11.8) | 54 m(median) | Age, gender, HBV, alcohol abuse, DM, cirrhosis, Child Pugh grade, ICG retension at 15 min, serum AFP, extent of hepatic resection, operative blood loss, any perioperative blood transfusion, tumor size, tumor no., any venous invasion, microsatellite lesion, tumor encapsulation, width of resection margin, histological margin involvement, pTNM staging. | Adjusted HR not given.  The unadjusted HRs were calculated based on original data:   - HCC recurrence-free survival: HR=0.92(95%CI: 0.52-1.64); - All-cause mortality in HCC patients: HR=0.95(95%CI: 0.65-1.39). |
| Huo TI, et al 2003 ^66^ | Taiwan | HC | Cancer registry Database of Taipei Veterans General Hospital 1996-1999, who underwent primary resection for HCC with complete medical history including serological markers for HBV and HCV and minimum follow-up of 6 mos. | 39/239  (16) | 32m (mean) | Age, sex, HCV, HBV, tumor size, number of tumors, cirrhosis, alcoholism, AFP, albumin, bilirubin, tumor stain, differentiation grade, operation. | All-cause mortality in HCC patients: (reference= Non-diabetic and HBsAg-positive HCC patients )   - RR=2.0(95%CI:1.1-4.1) |
| Huo TI, et al. 2003 ^65^ | Taiwan | HC | Taipei Veterans General Hospital 1996-1999, Child-Pugh Class A hepatic functional reserve at time of resection. | 40/245  (16) | 27m (mean) | Age, sex, HBV, tumor size, number of tumors, initial Child-Pugh score, liver cirrhosis, AFP, vascular invasion, tumor stain, histological differentiation, and operation method. | Occurrence of hepatic decomposition (increase in Child-Pugh of 2 or more points):   - RR=2.3(95%CI: 1.4-3.7). |
| Coughlin SS, et al.2004 ^63^ | USA | PC | ≥ 30 years  District of Columbia, and Puerto Rico, at least one household member ≥ 45 years old. | 52 803/1 056 243  (5) | 16y | Age, alcohol, race, years of education, BMI, smoking, total red meat consumption, consumption of citrus fruits and juices, consumption of vegetables, PA, hepatitis and cirrhosis | HCC specific mortality in men and women:   - Men: HR=2.19(95%CI: 1.76- 2.72); - Women: HR=1.37(95%CI: 0.94- 2.00). |
| Batty GD, et al.2004 ^64^ | Britain | PC | 40-65 years  Male government employees in London | 188/18 194  (1) | 25y | Age, employment grade, smoking, PA, systolic BP, BP-lowering medication, marital status, disease at study entry, unexplained weight loss, BMI, triceps skinfold thickness, height adjusted FEVI, plasma cholesterol | HCC specific mortality in men:   - Men: HR=4.74(95%CI: 0.59- 37.9). |
| El-Serag HB, et al. 2004 ^24^ | USA | HC | Department of Veteran Affairs records of hospitalized veterans registered within nationwide Patient Treatment File (discharge diagnosis since 1970) with DM discharge diagnosis between 1985-1990† | 17 3643/824 263 (21) | DM 8.6y (mean)  Non-DM 10.1y (mean) | Age, sex, ethnicity, period of military service, Charlson disease index. | HCC incidence (excludes new diagnosis of HCV, HBV, acute liver disease, alcohol use, and fatty liver in first year of follow up):   - RR=2.13 (95% CI: 1.79-2.53) |
| Huo TI, et al. 2004 ^62^ | Taiwan | HC | Taipei Veterans General Hospital April 1996-March 2001, 255 HCC patients who underwent surgical resection and 312 patients with non-resectable HCC (non surgical group) who underwent TACE and percutaneous infection therapy. | 120/567  (21) | Surgical patients:  33 m (mean)  Non Surgical patients: 24 m (mean) | Age, sex, HbsAg status, size & number of tumors, liver cirrhosis, child-Pugh class, Okuda staging, serum AFP, albumin & bilirubin levels, vascular invasion, tumor stain, tumor cell differentiation, and treatment method. | **Surgical Patients**:   - All-cause mortality in small HCC(<=5cm) group: RR=2.3(95%CI: 1.2-5.1);   - MV: age, AFP - All-cause mortality in free of tumor recurrence group: RR=1.7(95%CI: 1.1-2.6).   - MV: liver cirrhosis   **Non-surgical Patients:**   - All-cause mortality in Child Pugh Class A patients : RR=1.6(95%CI: 1.1-2.5).   - MV: multifocal tumors, tumor size >5cm - Hepatic decomposition in Child Pugh A: **RR =1.7(95% CI: 1.1-2.6)**   - MV: tumor size > 5cm |
| Jee S H, et al.2005^61^ | Korea | PC | 30 to 95 years  Received health insurance from The National Health Insurance Corp | 62 924/1 298 385  (4) | >10y | Age, age squared, alcohol, and smoking. | HCC specific mortality in men and women:   - Men: Not shown (overlap to Park SM, et al. 2006^60^) - Women: HR=1.28 (95%CI: 1.00-1.66).   HCC incidence in men and women:   - Men: HR=1.66 (95%CI: 1.53-1.79); - Women: HR=1.18 (95%CI: 1.00-1.43). |
| Park SM, et al. 2006^60^ | Korea | HC | ≥ 20 years  Men in The National Health Insurance Corp cohort with first cancer who participated in national health examination program starting in 1996. | 1223/14 578  (8.4) | 3.03 y(mean) | Age, alcohol, smoking history, insulin resistance before primary tumor, BMI, fasting glucose level, PA, cholesterol level, food preference. | All-cause mortality in male patients(DM was defined as fasting glucose ≥126mg/dl):   - HR= 1.04(95%CI: 0.89-1.22). |
| Lai M S,et al.2006^21^ | Taiwan | PC | >=30 years old residents in the Keelung Community-Based Integrated Screening program | 3 957/54 916  (7) | 2.78y(mean) | Age, sex, HCV, HBV, alcohol, and cigarette smoking. | HCC incidence:   - HR=1.84(95%CI: 1.10-3.07). |
| Khan M, et al.2006^22^ | Japan | PC | 40-79 years old healthy inhabitants from 45 municipal areas | 3 307/56 881  (6) | 8.05y(mean) | Age, alcohol, BMI, smoking. | HCC incidence in men and women:   - Men: HR=2.09(95%CI: 1.26-3.47); - Women: HR=2.55(95%CI: 1.07 - 6.10). |
| Inoue M, et al.2006^23^ | Japan | PC | 40-69 years  Registered Japanese inhabitants in the 10 public health center areas | 4 668/97 771  (5) | 10.7y(mean) | Age, alcohol, smoking, study area, history of cerebrovascular disease, history of ischemic heart disease, BMI, PA, green vegetable intake, and coffee intake | HCC incidence in men and women:   - Men: HR=2.24(95%CI: 1.64-3.04); - Women: HR=1.94(95%CI: 1.00-3.73). |
| Torisu Y, et al. 2007^20^ | Japan | HC | 1978-2003, 50 patients diagnosied as **having alcoholic cirrhosis**, heavy drinkers ( total alcohol intake over 500 kg), **negative HBsAg and anti-HCV antibody results**.  No occult HBV infection. | 11/47  (23) | 6.8 y (median) | Age, sex, total alcohol intake, history of cigarette smoking, family history of liver disease, history of blood transfusion, state of cirrhosis (presence of ascites and/or a history of encephalopathy), AST, ALT, albumin, bilirubin, globulin, AFP, platelet count and ICG R15. | HCC incidence:   - HR =21.7(95%CI: 2.4-193.7). - MV: Age |
| Komura T, et al. 2007^59^ | Japan | HC | Kanazawa University Hospital (1987-2004). Patients diagnosed with primary HCC and who underwent curative surgical treatment.^€^ | 30/90  (33) | >60m | Age, sex, HCV, BMI, prevalence of alcohol abuse, HbA1c, liver fibrosis degree, Child-Pugh classification, platelet count, ALT, total bilirubin, Albumin, AFP, tumor size, tumor differentiation degree, and presence of vascular invasion | HCC recurrence-free survival:   - HR=2.9(95%CI: 1.5-5.5). - MV: HbA1c, child pugh B classification. |
| Ioannou GN, et al, 2007^29^ | US | HC | Veterans Affairs Health Care Centers in North West US (1994-2005), patients who had a diagnosis of cirrhosis in medical records. | 447/2 126  (21) | 3.6 y (mean) | Age at diagnosis of cirrhosis, sex, race, HCV infection, HBsAg, HBV core antibody, alcohol abuse, cannabis abuse, HIV 1, BMI, serum bilirubin, creatinine, albumin, ALT, AST, ferritin level, PT INR, Hb, platelet count. | HCC incidence:   - HR=1.5 (95%CI: 0.9-2.5) - MV: Age at diagnosis of cirrhosis, HCV infection, HBsAg, HBV core antibody, DM II, BMI (obesity, overweight) low platelet count. |
| Kawamura Y, et al. 2008^57^ | Japan | HC | Toranomon Hospital, patients diagnosed with HCC from 1980-2006 with HCC associated with non-B and non-C hepatitis who later underwent surgical resection or RFA. | 18/40  (45) | 3.7 y(median) | Age, sex, alcohol history, smoking history, association of hyperlipidemia, history of blood transfusion, chronic hepatitis or liver cirrhosis, AST, albumin, bilirubin, AFP, DCP, PT activity, ICG-R15, tumor size, multiplicity, hypervascularity, and PV invasion. | HCC recurrence-free survival:   - HR= 4.61(95%CI: 1.53-13.88). - MV: Tumor size, blood transfusion, diabetes, prothrombin activity, serum albumin, total bilirubin, ICG-R15, and age |
| Di Costanzo GG. et al. 2008^28^ | Italy | HC | Patients referred to liver unit of Antonio Cardarelli Hospital from1994-2004.  Dcumented history of blood transfusion < 1990 and diagnosis of HCV related cirrhosis,  No HBV coinfection, any other concomitant causes of liver diseases (except alcohol), presence of HCC at first visit, history of IV drug abuse, or evidence of HIV. | 41/138  (30) | 7y (median) | Age, sex, alcohol [quantity, duration of consumption and total intake], smoking, earlier IFN therapy, diabetes. | HCC incidence in HCV related liver cirrhosis::   - RR=1.06(95%CI: 0.70-1.59). - UV |
| Veldt BJ, et al. 2008^27^ | Europe & Canada | HC | Patients with chronic hepatitis C and biopsy-proven advanced fibrosis or cirrhosis (Ishak score 4 to 6) treated between 1990 and 2003.  No HBV or HIV infection | 85/541  (16) | 4.0 y(median) | Age, anti-HBc positivity, fibrosis stage, DM, genotype, gender, BMI, bilirubin, albumin, and platelet count | HCC incidence:   - HR =2.07(95%CI: 0.95-4.47). |
| Ogunleye AA,et al.2009^19^ | Scotland | PC | Type 2 DM patients without any previous cancer and two matched non-DM comparators in the  population of Tayside Health Board registered with any Tayside GP practice | 9 577/19 154  (33) | DM: 1417d (mean)  Non-DM: 1476d(mean) | Deprivation (measured using deciles of a postcode score for material deprivation) and matched for age, sex and general Practice | HCC incidence:   - HR=3.50(95%CI: 1.38-8.91). |
| Oba S, et al.2008^58^ | Japan | PC | ≥ 35 years  Residents in Takayama City | 1 217/29 079  (4) | 6.9y(mean) | Age, alcohol intake, smoking, BMI, PA, years of education, history of hypertension, total energy intake, vegetable intake, fat intake | HCC specific mortality:   - HR=4.30(95%CI: 1.98-9.38). |
| Huo TI, et al. 2010^55^ | Taiwan | HC | Patients enrolled in Taipei Veterans General Hospital 2002-2008 with diagnosis of HCC. | 392/1 713  (23) | 18m (mean) | Age, sex, HBsAg status, anti-HCV, ECOG, tumor size, number of nodules, vascular invasion, serum AFP, serum creatinine level, CTP class | All-cause mortality:   - HR=1.2(95%CI: 1.02-1.42). - MV: Performance status (ECOG), tumor size, number of nodules, vascular invasion, serum AFP level, serum creatinine level, and CTP class. |
| Wang CS, et al.2009^18^ | Taiwan | PC | ≥ 35 years.  Residents in A-Lein Township | 546/5929  (9.2) | 8y(mean) | Age, sex, alcohol intake, smoking, HBV, HCV, HCV Genotype 1, BMI. | HCC incidence:   - HR= 2.7 (95%CI: 1.7-4.3) |
| Hung CH, et al.2010^26^ | Taiwan | HC | 16–78 years.  Patients with chronic HCV infection, treating with IFN or pegylated-IFN plus ribavirin therapy in a single center.  No decompensated liver disease, hepatitis B, autoimmune hepatitis or alcohol abuse, major contraindications to IFN or ribavirin therapy. | 253/1 470  (17) | 4.4y (median) | Age, sex, BMI, baseline cirrhosis, pretreatment platelet, sustained virological response, pretreatment AFP. | HCC incidence:   - HR =0.72(95%CI: 0.43-1.22) for both men and women - Men: HR=4.32(95%CI: 1.23-15.25) |
| Chodick G, et al.2010^17^ | Israel | PC | Men and women, with and without DM,  in a large health maintenance organization in Israel from the general population | 16 721/100 595  (16) | 8y(mean) | Age, region, SES level, use of healthcare services a year prior to index date, BMI, and history of cardiovascular diseases | HCC incidence:   - Men :HR=2.42 (95%CI: 1.00-5.84); - Women: HR=1.83(95%CI: 0.86-3.89). |
| Lam EK, et al.2010^56^ | Asia-Pacific | PC | ≥20 years.  Persons from 63 cohorts in general population | 23 560/367 361  (6) | 4y(median) | Age, sex, and study stratified | HCC specific mortality:   - HR=1.51(95%CI: 1.19- 1.91). |

Abbreviations: ALT,alanine transferase; AST, aspartic transaminase; AFP,α-fetoprotein; BMI, body mass index; BP, blood pressure, CI, confidence interval; CT, computed tomography imaging; CTP, Child-Turcotte-Pugh; d, day; DCP, des-gamma carboxyprothrombin; DM, diabetes mellitus; ECOG, Eastern Cooperative Oncology Group performance status; FEV1, forced expiratory volume in one 1 second; Hb, hemoglobin; HBV, hepatitis B virus; HbA1c, hemoglobin A1c; HbsAg, hepatitis B surface antigen; HC, hospital-based cohort; HCC, hepatocellular carcinoma; HCV, hepatitis C virus; HIV-1, human immunodeficiency virus 1; HR, hazard ratio; ICG-R15, indocyanine green retention rate at 15 minutes; IFN, interferon; INR, international normalized ratio; m, month; MRI, magnetic resonance imaging; MV, multivariate analysis; SES, socioeconomic status; PA, physical activity; PC, population-based cohort; PT, prothrombin time; PV, portal vein; RFA, radio frequency ablation; TACE, transarterial chemoembolization; UV, univariate analysis; y, year

‡Multivariate or univariate adjustments for each risk ratio reported if different than previously listed adjustements assessed.

^α^Criteria for surgery: absence of distant mets, IVC or main portal vein thrombosis, and anatomically resectable ds, and adequate liver function (Child Pugh classification, liver biochemistry, ICG clearance test)

^†^3 patients without DM randomly assigned and matched on year of hospitalization to each DM patient,

^€^ Criteria for surgery: good general condition, primary HCC, Child-Pugh classification A or B, number of HCC was solitary and no CT, MRI, or angiographic evidence of vascular invasion or distant metastasis.
